# Ag_2_S-Ag_2_O-Ag/poly-2-aminobenzene-1-thiol Nanocomposite as a Promising Two-Electrode Symmetric Supercapacitor: Tested in Acidic and Basic Mediums

**DOI:** 10.3390/mi14071423

**Published:** 2023-07-14

**Authors:** Mohamed Rabia, Asmaa M. Elsayed, Maha Abdallah Alnuwaiser, Ahmed Adel A. Abdelazeez

**Affiliations:** 1 Nanomaterials Science Research Laboratory, Chemistry Department, Faculty of Science, Beni-Suef University, Beni-Suef 62514, Egypt; 2 TH-PPM Group, Physics Department, Faculty of Science, Beni-Suef University, Beni-Suef 62514, Egypt; 3 Department of Chemistry, College of Science, Princess Nourah Bint Abdulrahman University, P.O. Box 84428, Riyadh 11671, Saudi Arabia; 4 Nanoscale Science, University of North Carolina at Charlotte, Charlotte, NC 28223, USA

**Keywords:** Ag_2_S-Ag_2_O-Ag/poly-2-aminothiophenol, nanocomposite, supercapacitor, acidic and basic medium

## Abstract

A Ag_2_S-Ag_2_O-Ag/poly-2-aminobenzene-1-thiol (P2ABT) nanocomposite was prepared using the photopolymerization reaction using AgNO_3_ as an oxidant. The size of the nanocomposite was about 40 nm, in which the morphology was confirmed using TEM and SEM analyses. The functional groups of Ag_2_S-Ag_2_O-Ag/P2ABT were confirmed using FTIR; also, XRD confirmed the inorganic Ag_2_S, Ag, and Ag_2_O formation. This nanocomposite has great performance in supercapacitor applications, with it tested in acidic (1.0 M HCl) and basic mediums (1.0 M NaOH). This pseudo-capacitor has great performance that appeared through the charge time in an acid medium in comparison to the basic medium with values of 118 s and 103 s, correspondingly. The cyclic voltammetry (CV) analysis further confirmed the excellent performance of the supercapacitor material, as indicated by the large area under the cyclic curve. The specific capacitance (C_S_) and energy density (E) values (at 0.3 A/g) were 92.5 and 44.4 F/g and 5.0 and 2.52 W·h·Kg^−1^ in the acidic and basic mediums, correspondingly. The charge transfer was studied through a Nyquist plot, and the produced R_s_ values were 4.9 and 6.2 Ω, respectively. Building on these findings, our objective is to make a significant contribution to the progress of supercapacitor technology through a prototype design soon.

## 1. Introduction

Under the increasing energy demand, renewable and sustainable energy devices have emerged as a promising solution to address this pressing energy challenge [1,2]. 

Electrochemical devices used in energy storage are characterized by a longer life and high energy and density, such as rechargeable batteries and rechargeable accumulators and supercapacitors [3,4,5]. These devices work in complete harmony from a technical point of view in terms of high energy density resulting from primitive capacitors and higher energy than accumulators for electrical welding applications [6,7,8]. To reach the best electrochemical performance, multiple electrolytes are used to create a voltage through charge separation for the cell and electrodes that are nano-sized and have a high porous structure [9,10]. To make the most of the electrochemical reactions, it is advisable to use electrodes with a relatively large specific porosity. The greater the capacity of the conductor to transport, the better the electrical conductivity. 

Supercapacitors have three categories for the way the charges are stored. The first type is double-layer electrochemical capacitors [11,12]. The second type of capacitors is pseudo-capacitors, in which the electrodes are subjected to mutual oxidation and reduction processes with the electrolytes [13,14]. The third category represents a mixture of these two types, in which the materials of the electrode are a mixture of these materials, carbon mixed with redox materials. Carbon materials can be found in extensive studies for their use as electrodes in supercapacitors to increase energy and density by depositing silver on them [11,15,16]. Recent studies have investigated the use of novel materials for supercapacitors, such as ZnO and ZnO/G-ZnO composites, which achieved a C_S_ of 61.7 F/g and 140 F/g, correspondingly [17,18]. Additionally, studies have explored the incorporation of Ag-Ag_2_O composite materials to enhance the performance of supercapacitors, highlighting their potential for use in these materials [19,20].

Polymer materials (semiconductors) [21,22] find their way into high technology devices related to energy storage and transfer, and they have great compatibility and stability, in addition to their low costs and availability [23,24,25]. These polymer materials are used in pseudo-capacitors, in which these polymers have a redox property. With these great advantages of polymer materials, there are several studies demonstrating using additive materials for enhancing energy storage. 

P2ABT has excellent optical and electrical properties for use in sustainable energy storage devices, particularly due to its ability to undergo redox reactions facilitated by its electronegative sulfur atom [26]. Additionally, its relatively simple synthesis process makes it an attractive option for industrial applications as it can be produced cost-effectively and efficiently [27,28,29].

Metal oxides consider the large and recommended materials for increasing energy storage through composites with polymer materials [30,31,32,33,34,35]. Silver ions work to increase the efficiency of transferring electrical charges and increase the electrochemical performance of materials [36]. The good conductivity of Ag works as an active site inside the composite for charge combination and then charge storage. The redox reaction of the composite causes these charge storages [37]. Kim et al. [38] illustrated the effect of silver in a composite, in which their study showed that silver nanoparticles increase the capacitance significantly, and also, they work to increase the graphite fibers’ charge and the electrochemical use of electrodes. Also, Atta et al. [39] studied a Ag_2_O/polyaniline composite in supercapacitor fabrication, in which there was great enhancement in the specific capacitance under the incorporation of this metal oxide with the polymer materials. 

The composite made of polymer materials with a metal oxide has a great property that combines both the advantages of these two materials, in which the polymer material increases the charge storage and the metal oxide with its stability increases the lifetime and stability of the synthesized supercapacitor. The ability of the polymer for charge storage may be related to the resonance phenomena, in which the polymer material has a great ability to accept additional electrons for redox reactions [37]. 

To our knowledge, no previous studies have examined Ag_2_S-Ag_2_O-Ag/P2ABT nanocomposites. Herein, the photopolymerization technique is demonstrated for the synthesis of a Ag_2_S-Ag_2_O-Ag/P2ABT nanocomposite from an acid medium (acetic acid), while P2ABT is synthesized using K_2_S_2_O_8_ as an oxidant. TEM, SEM, FTIR, and XRD analyses were performed to confirm all of the nanocomposite properties. This nanocomposite is applied as a paste for a supercapacitor from acid and basic mediums, in which the performance is greater in an acid medium. The electrochemical charge is demonstrated, in which the C_S_ and E parameters are measured. Moreover, the cycle voltammetry and impedance are investigated from both mediums. All the electrochemical parameters confirmed that the behavior of the fabricated pseudo-capacitor is greater in the acid medium. 

## 2. Experimental Section

### 2.1. Materials

The 2-aminobenzene-1-thiol, ethanol, and nafion (in methanol) were obtained from Merk (Darmstadt, Germany), VWR (Darmstadt, Germany), and Sigma Aldrich (St. Louis, MO, USA), respectively. The graphite powder, potassium persulfate (K_2_S_2_O_8_), acetic acid, and HCl were supplied by Pio-Chem Co., Giza, Egypt. 

### 2.2. Preparation of P2ABT and Ag_2_S-Ag_2_O-Ag/P2ABT

The P2ABT nanocomposite was prepared through the oxidation of 2-aminobenzene-1-thiol (0.12 M) using the oxidant K_2_S_2_O_8_ (0.15 M) and 0.5 M HCL. First, the monomer was stirred well in the presence of the acid medium; moreover, the oxidant dissolved well. Through the sudden addition of the oxidant over the monomer, the reaction was completed. During the polymerization reaction, a dark green precipitate represents the polymer deposition. This polymer was collected, purified, and dried well. 

**Figure 1:**
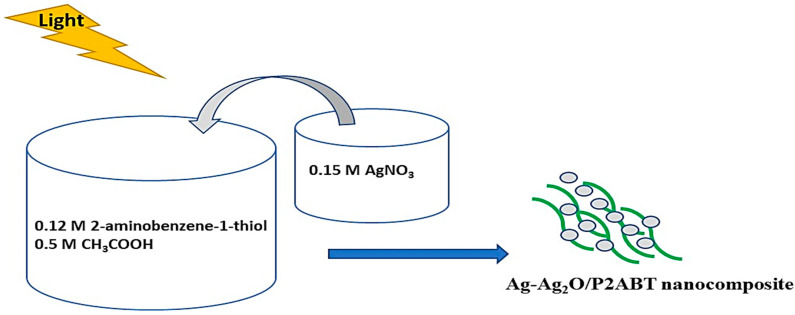
Schematic diagram of the photopolymerization reaction of 2-aminothiophenol to the Ag_2_S-Ag_2_O-Ag/P2ABT nanocomposite using AgNO_3_ as an oxidant.

On the other hand, the Ag_2_S-Ag_2_O-Ag/P2ABT nanocomposite was prepared through the photopolymerization of (0.12 M) 2-aminothiophenol using (0.15 M) AgNO_3_, in which acetic acid was used as the acid and solvent. Through this reaction, the Ag_2_S-Ag_2_O-Ag/P2ABT nanocomposite formed with its grayish-green color (Figure 1). 

### 2.3. Supercapacitor Fabrication

The fabrication of the supercapacitor was demonstrated through the loading of a paste made of the Ag_2_S-Ag_2_O-Ag/P2ABT nanocomposite into two Au plates with a 1.0 cm^2^ surface area. The paste was prepared through suspending the 0.04 g Ag_2_S-Ag_2_O-Ag/P2ABT nanocomposite in 0.005 g graphite powder, 100 µL nafion, and 750 µL ethanol; then, this paste (0.003 g) was loaded onto the two electrodes. Whatman paper saturated by 1.0 M of NaOH or 1.0 M of the HCl electrolyte was used. Then, the supercapacitor was closed well using adhesive tape. 

The electrochemical workstation (CHI608E) measured the supercapacitor’s performance through determining the CV and charges in a potential window from 0.0 to 1.0 V. Moreover, the stability and impedance were evaluated to determine the lifetime and charge transfer through the electrodes. Finally, the Cs, E, and power density (P) were calculated as an indication of the supercapacitor’s efficiency. 

### 2.4. Characterization

The characterization procedure took place to confirm the various morphological and structural characteristics of the manufactured materials. The chemical composition of the produced materials was verified using X-ray diffraction (X’Pert Pro, Almelo, Holland). SEM (ZEISS SUPRA 55 VP, Jena, Germany) and TEM (JEOL JEM-2100, Tokyo, Japan) analyses were evaluated for the 3D and 2D materials, respectively.

## 3. Results and Discussion

**Figure 2:**
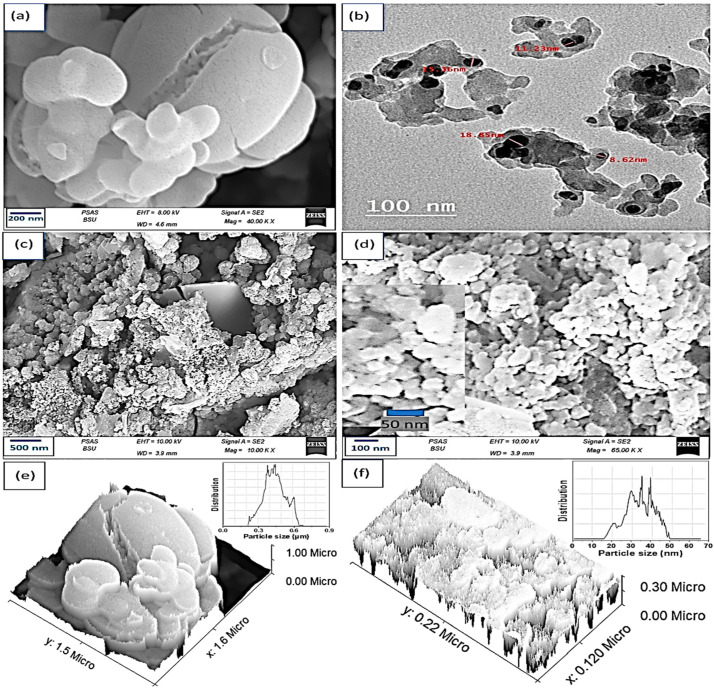
(**a**) SEM and (**e**) roughness modeling of the P2ABT. (**b**) TEM, (**c**,**d**) SEM, and (**f**) roughness modeling of the Ag_2_S-Ag_2_O-Ag/P2ABT nanocomposite.

The surface characteristics of the prepared P2ABT were determined through SEM analyses (Figure 2a). This figure suitably illustrates the formation of the broken ball-like-shaped P2ABT with a large surface area. This polymer has a particle size of about 120 to 800 nm; this confirms the formation of nano/micropolymers. The great surface area of this polymer motivates the composite formation through the reaction in the presence of additional materials [40,41,42]. 

The SEM images in Figure 2c,d showcase the morphology of the synthesized Ag_2_S-Ag_2_O-Ag/P2ABT nanocomposite at different magnifications. The nanocomposite exhibits nonuniform or semi-spherical particle structures, with the Ag_2_S-Ag_2_O-Ag particles well-coated within the polymer material. The average size of the nanocomposite particles was determined to be 40 nm. This morphology indicates a large surface area, which in turn suggests the presence of numerous active sites that facilitate efficient charge storage within the material. 

The TEM of the prepared Ag_2_S-Ag_2_O-Ag/P2ABT nanocomposite is demonstrated in Figure 2b. The formation of the nanocomposite was proven, in which the Ag_2_S, Ag, and Ag_2_O were present as dark colored materials (15 to 18 nm) embedded in the polymer materials (a faint gray color, about 40 nm). The great enhancement in the nanocomposite size related to the reaction with the Ag_2_S, Ag, and Ag_2_O materials under the construction of the Ag_2_S-Ag_2_O-Ag/P2ABT nanocomposite was demonstrated.

The cross-section and modeling studies for the P2ABT and Ag_2_S-Ag_2_O-Ag/P2ABT nanocomposite are illustrated in Figure 2e,f, correspondingly, in which the size distribution charts are inserted inside the figure. Great uniformity was observed for the nanocomposite with an average particle size of 45 nm, while the P2ABT had a nonuniform size distribution of 550 nm.

**Figure 3:**
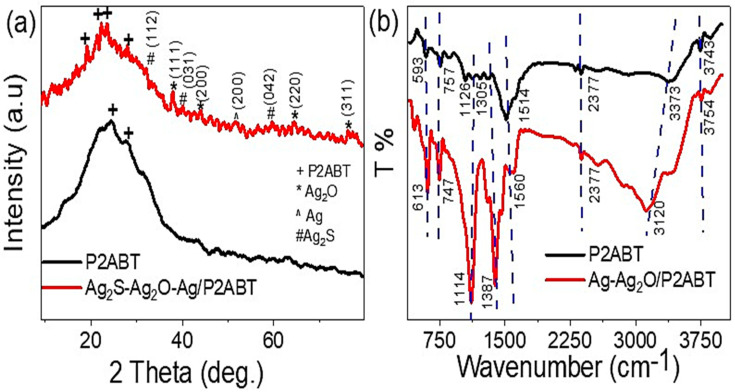
(**a**) The XRD and (**b**) FTIR for the prepared P2ABT and Ag_2_S-Ag_2_O-Ag/P2ABT nanocomposite.

The XRD pattern for the prepared P2ABT and Ag_2_S-Ag_2_O-Ag/P2ABT nanocomposite are shown in Figure 3a. The P2ABT (black curve) has an abroad peak, but there are two semi-sharp peaks located at 24.6 and 28.0; this behavior illustrates the formation of crystalline polymer materials [43]. 

After composite formation, resulting in Ag_2_S-Ag_2_O-Ag/P2ABT, there were great enhancements in the XRD pattern; this appeared through the formation of additional peaks characteristic of P2ABT and Ag and Ag_2_O nanomaterials. The P2ABT had four peaks located at 19.22°, 22.28°, 23.96°, and 27.94°. Moreover, the Ag_2_O materials had four peaks located at 38.05°, 43.99°, 64.67°, and 76.44° for the growth directions (111), (200), (220), and (311) for JCPDS 76-1393, respectively [44,45]. On the other hand, Ag_2_S appeared through the peaks at 32.3, 40.1, and 59.8 for the growth directions (112), (031), and (042) for JCPDS no. 14-0072, respectively [46]. While the Ag nanomaterial had one characteristic peak located at 39.54° for the growth direction of (200) for JCPDS NO. 04-0783 [44,47]. 

The FTIR of the P2ABT and Ag_2_S-Ag_2_O-Ag/P2ABT nanocomposite through the detection of the functional groups is illustrated in Figure 3b. All of the functional groups were confirmed well, in which the N-H and S-H vibration band values were located at (3743 and 3754 cm^−1^) and (3373 and 3120 cm^−1^), respectively [48]. The C-N group was present at 1126 and 1114 cm^−1^ after polymer formation. The C=C aromatic quinoid band values appeared at 1514 and 1560 cm^−1^, while the C=C aromatic benzenoid bands were located at 1305 and 1387 cm^−1^. The C-H in the plan values was at 1126 and 1114 cm^−1^. The shifts in the bands were due to the Ag_2_S, Ag, and Ag_2_O connections in the composite [49].

### The Electrochemical Study

**Figure 4:**
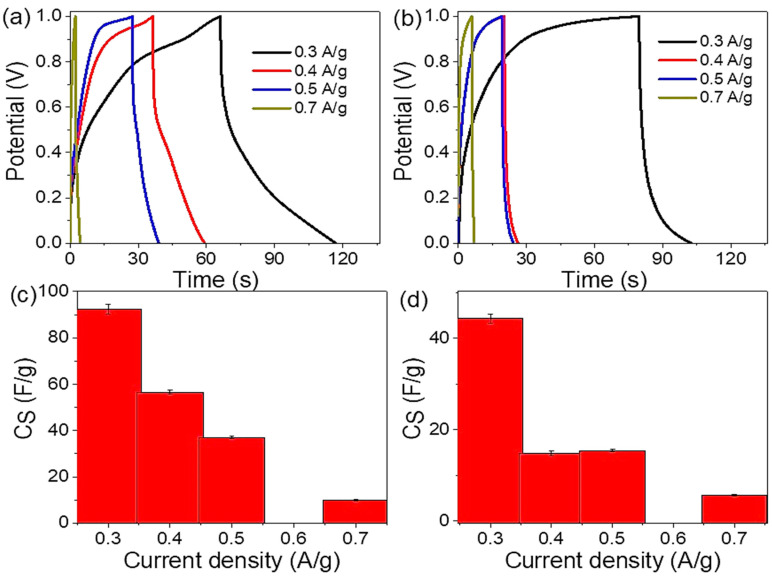
The electrochemical parameter (**a**,**b**) charge and (**c**,**d**) C_S_ values for the prepared Ag_2_S-Ag_2_O-Ag/P2ABT nanocomposite pseudo-capacitor under various electrolytes: acidic and basic mediums, respectively.

The charge/discharge study was performed through 0.3 to 0.7 A/g as illustrated in Figure 4a,b from the HCl and NaOH medium, respectively. The current density has a great effect on the charging behavior of the supercapacitor; the reverse relation of the current density with the charge storage appears clearly through the charge curves. Under high values of current density, the supercapacitor does not have sufficient time for charge storage [11,13,50]. There is greater enhancement in the charging behavior in the acid medium, in which the produced charge times are 118 s and 103 s. In addition to this greater time, the produced curve shows greater performance. This performance confirms the high mobility of the H^+^ ion and then the charge storage that motivates the role of the fabricated pseudo-capacitor; this matched with the previous literature [51,52], in which this ion has the ability as a proton jump that accelerates movement, and then, this ion has a great interaction with the composite for charge storage [53]. At the same time, the high porosity of the nanocomposite facilitates the penetration of these ions to its surface [54,55]. Despite OH^−^ (basic medium) having the same phenomenon of proton jumping, the basic nature effects on the polymer material decrease its conductivity [56], and then, the energy storage decreases. 

The specific capacitance (C_S_) of the Ag_2_S-Ag_2_O-Ag/P2ABT nanocomposite fabricated pseudo-capacitor is demonstrated through Equation (1) [57,58], in which the loaded mass is the main parameter in this equation, besides the potential windows (ΔV) that are applied on the electrodes and the produced discharge time (Δt). Through the observed values of C_S_ in Figure 4c,d, the produced C_S_ depends on the electrolyte medium of the fabricated pseudo-capacitor. In the acidic medium (Figure 4c), the C_S_ values decrease from 92.5 to 9.98 F/g with the current from 0.3 to 0.7 A/g, correspondingly. Through this current density range, the C_S_ values, for the basic medium (Figure 4d), decrease from 44.4 to 5.7 F/g. These results reflect the role of the acid medium for charge storage, which depends mainly on the H^+^ ion mobility through the proton jump phenomenon, while the basic medium reduces the conductivity of the P2ABT polymer, and then the produced Cs value is reduced. 

In the same way, the E values were calculated for the pseudo-capacitor in acidic and basic mediums. This calculation was applied through Equation (2) [57,58]; the square of potential windows was the main parameter through this equation besides the C_S_ values. The E values for the prepared supercapacitor were 5.0 and 2.52 W·h·kg^−1^ in the acidic and basic mediums, correspondingly, at a current density of (0.3 A/g). The power density (P) was illustrated using Equation (3) [13]. Moreover, the gravimetric capacitance (C_g_) was calculated using Equation (4) [12], using the values of the scan rate (s); these values are 0.9 and 0.74 F/cm^2^ in the acidic and basic mediums, correspondingly.

(1) $$C_{s}=4I\cdot \Delta t/\Delta V\cdot m$$

(2) $$E=0.5C_{s}\cdot (V_{max}^{2}-V_{min}^{2})/3.6$$

(3) P=3600 E/Δt

(4) $$C_{g}=\frac{4\int_{v_{1}}^{v_{n}}idv}{ms∆V}$$

**Figure 5:**
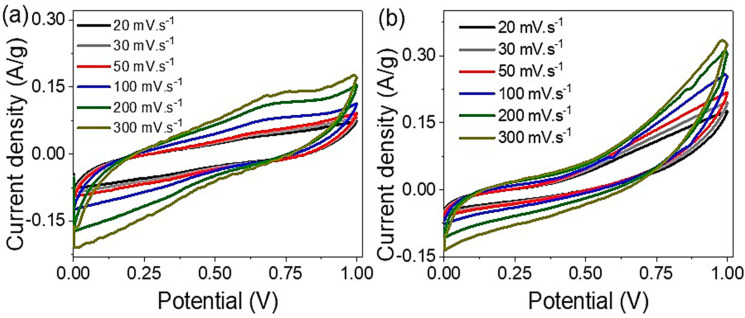
The cyclic voltammetry under the (**a**) acid and (**b**) base medium electrolytes for the fabricated Ag_2_S-Ag_2_O-Ag/P2ABT nanocomposite pseudo-capacitor.

The fabricated Ag_2_S-Ag_2_O-Ag/P2ABT nanocomposite pseudo-capacitor showed different behavior for the produced cyclic voltammetry study in the acidic and basic mediums as demonstrated in Figure 5a,b, correspondingly. When increasing the applied scan rate from 50 to 300 mV·s^−1^, the produced area under the cyclic curve increases well; this is related to the charge storage enhancements with scan rate values that are reflective of the redox reactions inside the supercapacitor [59,60]. From Figure 5a,b, the acid medium has a great effect on the enhancement of the charge storage, in which the produced cyclic curve is almost rectangular with a great area under the curve, while in the basic medium, the curve has a narrow area related to the small energy storage on the plates. 

**Figure 6:**
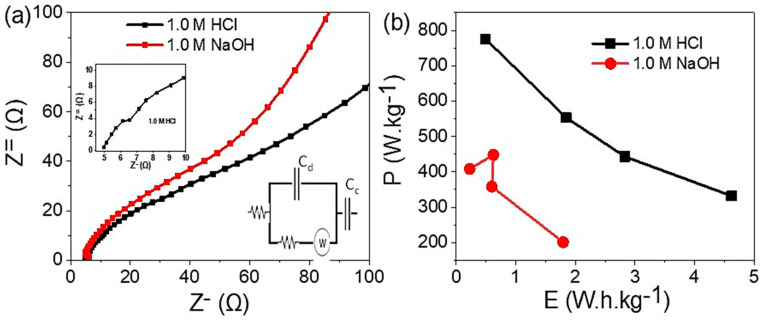
(**a**) The Nyquist plot for the fabricated Ag_2_S-Ag_2_O-Ag/P2ABT nanocomposite pseudo-capacitor and (**b**) the Ragone plot in the acidic medium (black curve) and basic medium (red curve).

The statement describes the charge transfer behavior of the Ag_2_S-Ag_2_O-Ag/P2ABT nanocomposite pseudo-capacitor (Figure 6). The charge transfer was evaluated by a Nyquist plot [14], which shows the real and imaginary impedance of the system. The Nyquist plot is represented by a black curve for the acid medium (1.0 M HCL) and a red curve for the base medium (1.0 M NaOH).

The Nyquist plot analysis reveals that the charge transfer process is more favorable in the acid medium (HCl) compared to the base medium (NaOH), as evidenced by the smaller semicircle observed in the former. This indicates that the presence of H^+^ ions facilitates the mobility of charges within the system. Furthermore, the acid medium enhances the conductivity of the polymer composite, thereby positively impacting its performance as a capacitor. This finding underscores the significance of the medium in which the charge transfer occurs and its consequential influence on the overall performance of the Ag_2_S-Ag_2_O-Ag/P2ABT nanocomposite pseudo-capacitor.

The statement further clarifies the charge transfer behavior of the Ag_2_S-Ag_2_O-Ag/P2ABT nanocomposite pseudo-capacitor by introducing the Randles circuit [11], represented in Figure 6a. The solution resistance (R_s_) and charge transfer resistance (R_ct_) of the system are illustrated from the Randles circuit. In the acid medium, the R_s_ and R_ct_ values of the Ag_2_S-Ag_2_O-Ag/P2ABT nanocomposite pseudo-capacitor were found to be 4.9 and 1.5 Ω, respectively. This is indicated by the small semicircle observed in the Nyquist plot. On the other hand, in the basic medium, the R_s_ and R_ct_ values are 6.2 and 1.6 Ω, respectively. The results obtained from the Randles circuit analysis support the conclusion that the acid medium facilitates the charge transfer process in the system. This is consistent with the findings from the Nyquist plot analysis. In summary, the Randles circuit analysis provides further evidence to support the role of the acid medium in enhancing the charge transfer performance of the Ag_2_S-Ag_2_O-Ag/P2ABT nanocomposite pseudo-capacitor. Under the great charge transfer of the nanocomposite using the HCl electrolyte, a magnified curve is inserted in Figure 6. The Ragone plot [61] indicates the greater enhancement of the power energy (P) in the acid medium (Figure 6b).

**Figure 7:**
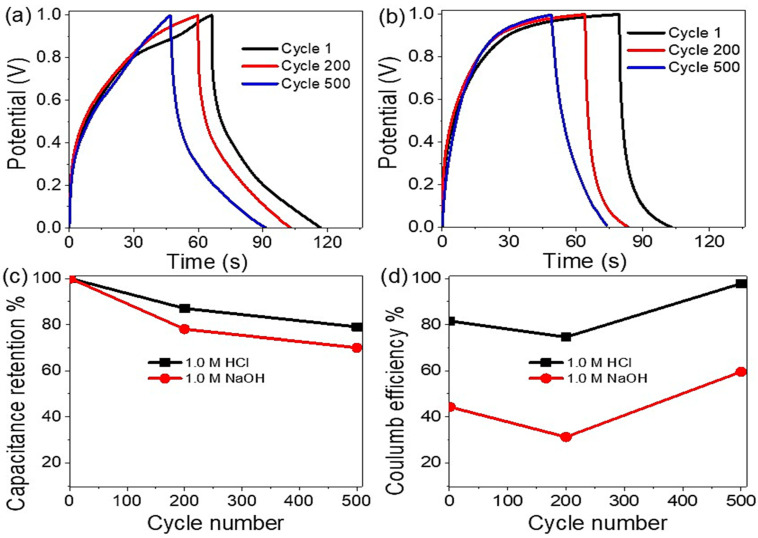
The stability of the fabricated Ag_2_S-Ag_2_O-Ag/P2ABT nanocomposite pseudo-capacitor under (**a**) acid and (**b**) base mediums; (**c**) the capacitance retention; and (**d**) the coulomb efficiency for this pseudo-capacitor.

For the fabricated Ag_2_S-Ag_2_O-Ag/P2ABT nanocomposite pseudo-capacitor’s stability, its performance was determined through an electrochemical charge study until 500 cycles from the acidic and basic mediums, Figure 7a,b, correspondingly. At 0.3 A/g, from these figures, the medium has a great effect on the stability of the fabricated pseudo-capacitor. The supercapacitor has retention stability values of 87% and 79% in the acidic and basic mediums, respectively, until 200 cycles. The capacitance retention and the coulomb efficiency (ɳ, using Equation (5)) [62] under these different electrolytes are represented in Figure 7c,d, correspondingly. This good stability under an acid medium confirms the conductivity enhancement of the polymer under the effect of H^+^ ions on the prepared polymer materials [13,63]. For additional information, we compared the efficiency of this fabricated supercapacitor with previous studies as mentioned in Table 1.

(5) ɳ = (Discharge time/Charget ime) ∗ 100

## 4. Conclusions

A highly efficient Ag_2_S-Ag_2_O-Ag/P2ATH nanocomposite with a small particle size (around 40 nm) was synthesized through a photopolymerization reaction and characterized using various analytical techniques. XRD and FTIR analyses confirmed the Ag_2_S, Ag, and Ag_2_O nanomaterials’ formation, while morphological analyses confirmed their diffusion inside the P2ABT polymer. The resulting nanocomposite was applied on both electrodes of a pseudo-capacitor, which was then tested in both acidic and basic mediums. The charge/discharge time was 118 s and 103 s for the acidic and basic mediums, respectively. The efficiency of the device was determined through its C_S_ and E values (at 0.3 A/g), which were 92.5 and 44.4 F/g and 5 and 2.52 W·h·Kg^−1^ in the acidic and basic mediums, correspondingly. The Nyquist plot (impedance value) and Ragone plot (energy and power density values) confirmed the superiority of the acid medium for the charge storage inside this supercapacitor. Due to the excellent properties of the device in an acidic medium, our team is currently working on developing a low-cost and easily fabricated prototype for industrial applications.

## Figures and Tables

**Table 1:** Comparison of the current work with other studies for supercapacitor performance.

Supercapacitor Material	Used Electrolyte	Current Density A/g	Capacitance (First Cycle) (F/g)	Capacitance (500th Cycle) (F/g)
Ppy/metal composite [64]	poly(vinyl alcohol)/H_3_PO_4_	0.005	70	50
CaO/G-C3N4 [65]	6 M NaOH	0.5	84	--
NiO/nanowalls [66]	1 M KOH	-	-	--
G-C3N4 [67]	1 M NaOH	1	20.5	19
β-Ni(OH)_2_ [67]	1 M NaOH	1	14.2	13.1
Carbon nanotube/Ag [68]	Gel electrolyte (polymethyl methacrylate, acetone, H_2_O, H_3_PO_4_)	0.001	88	--
Ag_2_S-Ag_2_O-Ag/P2ABT (current work)	1 M HCl	0.3	92.5	74

## Data Availability

The data will be supported up on request.
